# Assessing the impact of humanitarian assistance in the health sector

**DOI:** 10.1186/1742-7622-1-3

**Published:** 2004-10-07

**Authors:** Les Roberts, Charles-Antoine Hofmann

**Affiliations:** 1 Humanitarian Policy Group, Overseas Development Institute, London, United Kingdom

## Abstract

There have been significant improvements in the design and management of humanitarian aid responses in the last decade. In particular, a significant body of knowledge has been accumulated about public health interventions in emergencies, following calls for developing the evidence base of humanitarian health interventions. Several factors have prompted this, such as the increased volume of humanitarian assistance with subsequent higher levels of scrutiny on aid spending, and greater pressure for improving humanitarian aid quality and performance. However, documentation of the ability of humanitarian interventions to alleviate suffering and curb mortality remains limited. This paper argues that epidemiological studies can potentially be a useful tool for measuring the impact of health interventions in humanitarian crises. Survey methods or surveillance systems are mainly used for early warning or needs assessment and their potential for assessing the impact of aid programmes is underutilised.

## Introduction

In the last decade, humanitarian aid agencies have put a great deal of effort into improving the design and management of humanitarian responses in conflict situations: humanitarian agencies have agreed on international standards, numerous technical guidelines have been produced, training programmes are offered on different aspects of humanitarian programming, and aid programmes are evaluated more systematically. Initiatives as distinct as the Code of Conduct, Sphere, Active Learning Network for Accountability and Performance in Humanitarian Action, the Humanitarian Accountability Partnership International, the Quality Project from the French group Urgence, réhabilitation, développement and People in Aid all have in common a concern for the quality, performance and impact of humanitarian assistance [1]. In particular, a substantial body of knowledge has been accumulated regarding public health interventions in emergencies, in response to calls for developing the evidence base of humanitarian health interventions [2-4]. Most of these developments have been stimulated by the findings of the system-wide Rwanda evaluation, which highlighted serious questions about the performance of some humanitarian organisations and, more importantly, emphasised the responsibility of the international community: humanitarian aid alone cannot substitute for political action [5,6].

Despite the technical progress in the last decade, knowledge of the impact of humanitarian interventions in alleviating suffering and ultimately reducing mortality in the health and other sectors remains limited. There is a real question whether all the developments in accountability and implementation actually improve the overall performance of humanitarian assistance [7,8]. Whereas there is a significant evidence base on the effectiveness of interventions in acute emergencies, especially in refugee settings, the evidence base is much weaker for situations of protracted conflict with longer term programmes in less controlled settings. It is also difficult to determine the relative contributions of humanitarian aid programmes as distinguished from local coping mechanisms and/or the interventions of national governments. This lack of knowledge exists at the programmatic level and more generally at the sector-wide level.

This paper examines the current practice of humanitarian agencies for measuring the impact of health interventions. It is based on a review of the literature as well as on the authors' own field experience. The question explored is whether field epidemiology provides a useful set of tools and methods to determine more accurately the impact of health interventions.

## The impact of humanitarian assistance

The issue of impact is particularly high on the current humanitarian agenda. The increasing interest in impact analysis arises from a number of interlinked developments: the rapid increase in the overall volume of humanitarian assistance in the last decade- from 2 billion in 1990 to $5.5 billion by 2000 [9]- and the resulting scrutiny on how money is spent, the new public management agenda within the public sector and the adoption of results-based management systems by donors and some aid agencies.

There is no definition of impact in humanitarian assistance. The most commonly used definition of impact in international aid is the one provided by the OECD/DAC which defines impact very widely as ' [t]he positive and negative, primary and secondary, long-term effects produced by a development intervention, directly or indirectly, intended or unintended' [10]. This paper concentrates on the intended effects of aid interventions, e.g. whether the original objectives of a programme have been met. The question of negative, unintended impact, which is undoubtedly important, falls out of the scope of this paper.

A central question in an impact assessment is 'what would have happened in the absence of the aid programme?' In theory, there are two approaches to answering this question [11]:

1. To compare the impact with a control group that did not receive aid – a "with/without" comparison.

2. To do a "before/after" comparison for the beneficiaries of an intervention.

These two approaches pose different sets of issues. The "with/without" comparison with the use of control groups creates ethical problems: it is difficult to deliberately exclude a group from access to potentially life-saving relief. It may happen that some particular groups do not receive relief, due to problems with access or lack of resources, but, as Hallam warns, comparisons between people who received assistance and those who did not need to be used very carefully [12]. As a result, very few experimental trials with randomised allocations of services have ever been conducted in emergency or refugee situations [13,14].

The "before/after" comparison generates different problems. In order to be valid, the comparison implies that no other factor influences the impact, so that it can be fully attributed to the intervention. In reality, a multiplicity of other factors have an influence on the impact, such as the presence of other aid programmes, local coping mechanisms, or changes in the social and economic environment. For example, it is very difficult to attribute a reduction of conflict-related excess mortality to humanitarian aid only: variations in the baseline mortality (due to seasonal trends, disease epidemics, HIV/AIDS etc.) can be significant. There is an increasing recognition of the importance of these other factors, and that humanitarian aid, however vital, is only one element of the picture [15-18].

## A theoretical model for measuring impact

There are three main considerations that are necessary for measuring the impact of health programmes: the strength of the evidence suggesting causation between the intervention and the change in health status, the validity of a baseline of comparison, and the validity of the indicator employed. These conditions also apply to other types of humanitarian assistance.

### Criteria of causation

Over time, a great deal of debate has arisen over what epidemiological evidence constitutes proof that some exposure or input produced an effect versus what evidence simply implied an association. This distinction can be more than academic, as was seen over three decades of debate regarding the effects of smoking on health. Bradford-Hill put forward criteria for attempting to ascribe causation between an exposure and a health outcome [19]. These criteria are so widely utilised that some introductory text books simply refer to them as the epidemiological criteria of causation [20]. While his main motive was to attribute causation of a disease due to exposure to a chemical or biological agent, the logic of these criteria also applies to assessing the positive effects of favourable exposures, such as health programmes.

Bradford-Hill said that all of the following conditions can contribute to the argument that an exposure induces a health consequence:

1. The greater the strength of the association, the more likely that it is causative

2. There is a dose-response relationship between the exposure and the health outcome

3. Exposure consistently induces the health consequence in different settings at different times

4. The exposure occurs before the health outcome

5. There is a biologically plausible explanation for the exposure resulting in the health outcome

6. There are not more plausible explanations for the health outcome

7. Experimental results add particular weight to the evidence

For virtually all cause and effect health relationships, some of these criteria will not apply. For a programme to be shown to have an impact, criteria 4, 5 and 6 should always be met. Of particular concern to programme evaluation is the issue of biological plausibility and the amount of service provided. Programmes need to be evaluated with particular regard to the likelihood that the level of inputs provided could plausibly result in the outcome reported. That is, the number of clinic visits, or the amount of food provided per child etc. need to be sufficient to induce the health effects observed. Criteria of causation are important for interventions and settings where project impacts are usually not, or cannot be, measured.

These criteria of causation can be applied to populations and programmes as readily as Bradford-Hill applied them to specific disease agents. For interventions with a vast literature documenting the attributable benefits (e.g. measles vaccination or Vitamin A supplements), the need to show "proof" that the intervention produced a health benefit may be small, but for many other emergency interventions (e.g. HIV prevention through educational efforts or health benefits from shelter) there may be little or no evidence that such programmes produce any health benefits, making the importance of documenting any benefits great. Most humanitarian programmatic efforts fall somewhere in between, employing types of programmes that have produced documented benefits in some settings, but have failed in others, and may or may not be producing benefits in the setting at hand. Table 1 provides an example of how Bradford-Hill's criteria can be used.

**Table 1:** Application of Bradford-Hill criteria: Katana, Democratic Republic of Congo

**Figure 1:**
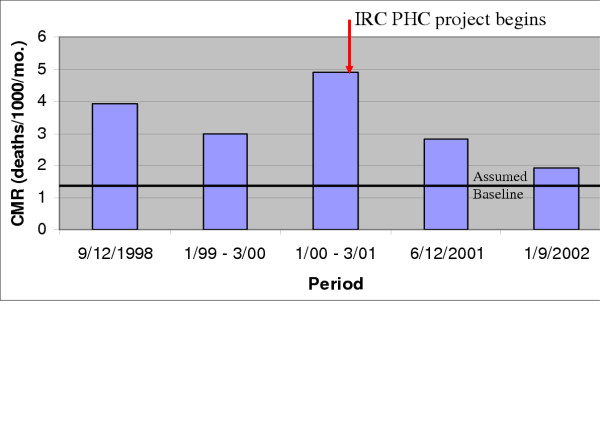
Mortality in Katana, 1998 – 2002

Starting in December of 2000, the International Rescue Committee (IRC) began a general health programme to support existing government services in Katana Health Zone, Democratic Republic of Congo (DRC). The IRC conducted population-based mortality surveys in this area with 345,000 mostly rural residents. The programme consisted of the provision of drugs, supplies, training and medical oversight in the clinics, a water provision and hygiene education programme in villages with the highest rates of cholera in 2000, a measles immunisation and vitamin A provision campaign, and support to the local health committees which included the donation of vouchers for the most indigent community members. Figure 1 below shows the crude mortality rate (CMR) over the period covered by 5 surveys conducted between 1999 and 2002. IRC claims to have reduced the excess CMR by 60% (from 4.9 to 2.8 deaths per 1000 per month where the baseline is assumed to be 1.5) during the period from 6 to 12 months after implementation and by 70% (from 2.8 to 1.9 deaths per 1000 per month) over the period from 12 to 24 months after implementation. In support of the results in figure 1 being a consequence of the health programme, IRC reported that:
• attendance at the clinic rose by 147% between 1999 (~7400 visits per month) and 2001 (~18,300 visits per month average)
• 70% of treatments were for malaria and diarrhoea, the main reported causes of death in the 1999 and 2000 surveys, and decreased as a cause of death in 2001 & 2002
• CMR in the five eastern provinces of DRC was estimated by IRC to have increased slightly in 2001 compared to 2000
• A survey in November of 2001 found that 60% of residents that had experienced fever in the preceding two weeks had sought treatment at a clinic
Employing Bradford-Hill's criteria, this example shows that: 1) there was a considerable drop in CMR associated with the establishment of the intervention, 2) there was no dose-response effect, 3) the fact that IRC's two other areas of health programmes had similar (but somewhat less dramatic) reductions implies repeatability, 4) the benefit occurred after implementation, 5) the findings are biologically plausible (although 1 visit per resident per year seems low), 6) alternative explanations for the reductions cannot be ruled out given the variance over time and the dramatic changes in violent conflict, although IRC reports that the violence did not dramatically subside until 2002, 7) these are not experimental data. Finally, the fact that the CMR was measured by an apparently valid survey method implies that IRC probably did contribute to a reduction in mortality in Katana [26].

#### Validity of the baseline of evaluation

Attempts to analyse the impact of humanitarian interventions are often handicapped by a lack of baseline data and a lack of knowledge about regular seasonal variations in key indicators of impact. For instance, baseline mortality rates are often not known. Countrywide figures are either unreliable, out of date, or not appropriate as they do not capture the regions where the conflict is occurring. A related problem that is continuously faced by humanitarian agencies is the lack of reliable population statistics [21].

When there are no baseline data, established norms can be used instead. For example, when people are arriving in a new location or are returning home, it is often impossible to determine the baseline before their arrival. In those cases, norms can be applied as an assumed baseline or as a threshold above or below which the indicator should not fall. Programmes lacking baseline data which keep mortality "low" or keep water and food provision high may be successful in terms of meeting their objectives but still not be able to quantify the impact of the intervention.

#### Validity of the indicator employed

The identification and use of relevant indicators is a crucial part of determining the impact of an intervention. Although the terminology varies, the literature generally distinguishes between performance (or process) indicators and impact (or outcome) indicators.

• Performance (or process) indicators concerns both the outputs of a programme (number of latrines built, number of training conducted, the quantities of food delivered) and the process of implementation (coverage of a programme, equity of distribution, targeting)

• Impact (or outcome) indicators are measures of the actual achievements intended by a programme. Mortality and malnutrition rates are the most commonly used impact indicators for humanitarian programmes, although interventions that aim to support livelihoods as well as save lives might require a broader set of indicators

For example, in a measles immunisation campaign, the immunisation coverage within a certain age group is a performance indicator, and the incidence rate of measles cases within that group is an impact indicator [22].

The Standardised Monitoring and Assessment of Relief and Transition (SMART) inter-agency initiative is an attempt to systematise the collection of impact indicators. SMART has recently stipulated that two measures (Crude Mortality Rate and nutritional status of children under five) are the most basic essential indicators for assessing the severity of population stress and for monitoring the overall effort of the humanitarian community. Those involved in the SMART initiative are currently developing standardised survey methodologies with associated reporting formats and software to address some of the challenges to effective evaluation. Part of the purpose of SMART is to enable global comparisons about the extent of humanitarian need in order to enable resources to be focused where they are most needed. A better understanding of needs based on nutrition and mortality might also enable better analysis of impact. Of course, malnutrition and mortality rates will not necessarily enable impact to be attributed to particular projects or agencies and may only be able to demonstrate the extent to which a relief system as a whole is meeting the needs of a population [23].

There is a tendency for humanitarian agencies to collect performance rather than impact indicators. This is due to various reasons, such as donor requirements that tend to favour the collection of performance indicators or the belief that impact indicators (such as mortality or morbidity) are sometimes difficult to collect. Arguably, it is easier for humanitarian agencies to monitor their own activities than to go out and monitor or assess the effect these activities have on the population they are assisting. Performance indicators may in some cases provide sufficient evidence about the likely impact of interventions and hence be used as a proxy for impact. However, when the validity of the proxy measure is unclear, there are risks in using performance indicators as a measure of success. For example, there is strong evidence that immunising children against measles has a direct effect on reducing mortality from measles; therefore, immunisation coverage can be used as a proxy for impact on mortality [24,25]. Other types of health interventions, such as reproductive health care, require more research on the link between the intervention and the health outcome before performance indicators can be used as a proxy for health outcomes [24]. Table 2 illustrates some commonly used indicators with regard to their strength of association to health outcomes, and the ease with which they can be monitored.

**Table 2:** Characteristics of indicators commonly used to justify health programmes.

**Established validity as measure of health impact**	**Indicator**	**General ease of acquiring data to show health effects**
Highest	• Crude Mortality, <5 mortality	Difficult in rural/diffuse settings, easier in camps
	• Case fatality rate	
High	• Nutritional status of children	Easy at the clinic data level, difficult but more valid with population surveys
	• Disease rates	
	• Immunisation status of children	
	• Patient-specific mental health evaluations	Logistically easy, requires skill on part of evaluator
	• Safety of blood supply	
Moderate	• Food-basket evaluations	Easy in camps, more difficult in more diffuse populations
	• Water and sanitation availability	
	• Reduction in measles, mumps and rubella through reproductive health services	Very difficult to measure even though benefits are likely to be occurring
	• Improved patient outcomes via referrals	
	• Impregnated bednets distributed	
	• Comprehensive, timely health information system	Nearly impossible. These are difficult to measure, and all require a series of events to induce a health benefit
	• Good coordination between sectors	
	• Knowledge & attitudes about services available	
	• Population practices	
Low	• People given seeds and tools, shelter, or other materials	Easy to measure. Links to health are likely to be mediated via many steps.
	• Drainage, fly control activities or tasks	
	• Number of clinic visits	
	• Distance to facilities, health workers per capita	
	• Trainings conducted, numbers trained	Easy to measure. May produce no effects on health.
	• Change in knowledge without documented change in behaviour	
	• Messages/curricula developed	

#### Current practice of humanitarian agencies in the health sector

In the humanitarian sector, assessment of impact has most often been seen as a sub-set of evaluation. Impact is one of the Organisation for Economic Co-operation and Development / Development Assistance Committee (OECD/DAC) evaluation criteria [4,27]. However, current evaluation practice rarely provides sufficient time for proper impact assessments. Most evaluation reports reviewed for this paper do not go beyond making statements about the impact of interventions.

Whereas the question of impact is unarguably important in evaluation practice, a detailed analysis of impact requires a different form of investigation. This may be done either through ongoing monitoring of project implementation, or as a separate research exercise (through surveys, operational research, reviews etc.). The tools developed in field epidemiology provide a set of potentially useful methods for measuring the impact of interventions. Existing approaches such as survey methods or surveillance systems, although seldom used by humanitarian agencies, can provide significant insight about the impact of humanitarian aid. So far, these tools remain mainly used in early warning systems or needs assessment [21]. The two most common approaches are survey methods and surveillance systems.

#### Survey methods

Most surveys are an attempt to actively go out and survey a representative sample of the population, although they may take different forms. WHO and others have produced manuals to guide health workers to conduct specific kinds of surveys, with nutritional anthropometry and Expanded Program on Childhood Immunizations (EPI) coverage methodologies being among the most succinctly described. Aid workers often do not have sufficient skills to take a valid sample and analyse the results of a survey. This is why many initiatives to improve the quality of relief programmes have emphasised the importance of training relief workers in survey methodologies. Some organisational headquarters and some groups such as Epicentre have specialists who can be deployed to assist with the conducting of surveys.

Uncertainty about population figures creates particular difficulties in constructing sampling frames for use in surveys. Census data may be many years old while the crisis may have had a dramatic impact on demographics and population numbers due to migration and high mortality. Although cluster surveys are a compromise measure, in many situations (especially in conflict situations or where terrain is very difficult) it may prove difficult to gain access to the 30 clusters proscribed. Nomadic groups may also prove difficult to sample [23,28,29]. Despite these difficulties, recent experiences have shown that surveillance methods can be successfully carried out in volatile environments [26].

#### Surveillance Systems

Surveillance is the systematic collection of information over time for decision making. Surveillance systems are often part of general monitoring systems and have been used for analysing impact in both health and nutrition programmes.

Aid agencies sometimes evaluate health programs by establishing a surveillance system at the beginning of a funding cycle and contrasting the rate of health events at the beginning and the end. This is valid if either: a) all of the events of interest are captured by the surveillance network, or b) the data from within the system are representative of the health conditions of the entire population and remain consistently so over the course of the project. Neither of these conditions is commonly met for clinic-based surveillance systems in rural and urban areas, although both of these conditions are often met in well-defined settings like refugee camps. If the majority of a population does not have access to formal health care then a clinic or hospital-based surveillance system will be able to tell very little about the health conditions of the broader population. Of course, not all surveillance systems are linked to utilisation of formal health services. Sentinel site surveillance systems for nutrition monitoring, for instance, involve the monitoring of purposively selected communities in order to detect changes in context, programme and outcome variables. Surveillance systems can be less costly than surveys and may reveal more in-depth information on the causes of malnutrition.

#### Problems with the lack of skills

A recurrent problem with the use of epidemiological methods such as surveys and monitoring is the lack of appropriate skills for conducting good quality assessments. Reviewers from the Centers for Disease Control and Prevention (CDC) evaluated the monitoring of projects and the measurement of nutritional status and mortality in Somalia from the period 1991–93 [30]. They developed a set of criteria for evaluating different kinds of information (surveillance and surveys) and systematically reviewed available reports. They found that the range of methodologies employed and outcomes measured were so variable and of such poor quality that they prevented widespread comparisons, and that, regardless of consistency, much of the data were simply not credible due to poor collection methods. Spiegel et al. from CDC reviewed 125 nutritional surveys conducted in Ethiopia in 1999 and 2000 during a time of famine but relative peace and stability [31]. The surveys were carried out by 14 organisations with a wide range of survey expertise. Only 67 of the 125 surveys attempted to conduct a sample that represented the population served. Only 9 of those 67 surveys assigned clusters to the population in a manner that was proportional to the sub-units of the population and only 6 of those possessed the minimum number of clusters (30) and children (900) suggested by most nutritional manuals. Spiegel concluded that non-governmental organisation (NGO) workers were woefully unprepared to conduct quantitative assessments of this kind, and that most of the surveys were of such poor quality as to be unhelpful toward making sound relief policy decisions [31].

The measurement of anthropometry is relatively standardised compared to many other health outcomes such as mortality and mental health status. For example, a mortality survey in Kabare Health Zone in the Eastern Democratic Republic of Congo (DRC) in 1999 was conducted simultaneously with an EPI coverage survey and only included households with a child under 5 years of age. The resulting estimate of crude mortality (1.9 per 1000 per month) was far lower than in a later repeat survey (finding 2.7 per 1000 per month) and, in fact, the initial survey missed most of the excess mortality [32]. For some project objectives, such as the prevention of HIV transmission, there is not even a widely agreed upon outcome to be measured that serves as a proxy for HIV incidence. The difficulty of assessing outcomes such as mortality is a principal reason for the use of process indicators in place of health outcomes.

Thus, without improved staff skills and capacity and a significant change in attitude among donors, it is likely that humanitarian agencies will continue to rely heavily on process indicators and not be expected to prove that programmes influenced the health of the targeted beneficiaries.

#### Review of 15 reports of health-related programmes

All final reports of health-related programmes funded by the US Department of State, Bureau of Population, Migration, and Refugees (BPRM) and submitted in 2003 were reviewed for the Humanitarian Policy Group study. Proposals that contained objectives of health-related activities (e.g. shelter provision, food transport) but that did not specifically say they would influence health status were excluded. The remaining 15 final reports were evaluated against the following five criteria:

• Was there a health-related objective?

• Was the baseline rate measured or a comparison group identified?

• Was the health-related outcome measured and reported?

• Was the societal level of the evaluation appropriate given the intervention?

• Were there any major issues supporting or raising concerns about the reported outcome data?

The societal level of a health project and evaluation was categorised as being either on the patient level, the household level or the community level. The expectation was that programmes that intervened on a specific level should be evaluated on that level. For instance, a curative health programme might have benefits at the individual level but it may not be possible to evaluate its impact at a wider level.

Six of the 15 reports did not attempt to measure or report any health-related rates or status. Proposals corresponding to five of these six reports only contained process indicators as the initial objectives, and thus the lack of documented health benefits was assured before the projects began. An additional three of the 15 reports contained health data-based objectives but did not present any health-status data, instead reporting process indicators such as the numbers of clinics supported, consultations given, or tons of food distributed.

Only four of 15 final reports could demonstrate a health benefit, and three others were likely to have produced a population-based benefit although this was not documented. These four were the only projects to measure baseline rates. Nine of the 15 did not have objectives and measures that matched to societal level. The results of this analysis confirm the general conclusion reached at the July 2002 SMART Monitoring and Evaluation Workshop, that while NGO's and agencies often want to monitor health outcomes, they usually monitor process indicators. Problems with process indicators seen in the BPRM review include:

• The cited activity may be related to the health outcome, but the significance of this effort depends on the activities being done well and in sufficient numbers (e.g. Eritrea and Sierra Leone wanted to reduce mortality and reported numbers of clinic-based activities)

• The health-related objective is only distantly related to the health outcome (e.g. a programme in Uganda wanted to induce "food self-sufficiency" but reported tons of food distributed)

• In some cases, the link between the process indicator and the outcome was simply implausible (e.g. a Balkans programme wanted to reduce dependency on aid of chronically "Extremely Vulnerable Individuals" and reported doing this for some by distributing school books)

Interestingly, a mental health programme in Guinea, with perhaps the most difficult-to-measure outcomes, had the most rigorous documentation, which included pre-intervention and post-intervention patient evaluations and the use of non-patient controls. Representatives for the other three programmes which documented impacts felt that very little of the project budget (perhaps <2%) was spent on documenting the impacts.

Over 20 NGOs were providing general health services in the eastern DRC in 2000 and 2001 with funding from either Office of U.S. Foreign Disaster Assistance (OFDA) or European Commission's Humanitarian Aid Office. According to OFDA, only two of those agencies could show health benefits associated with their programmes [33]. This seemed plausible at the time given the violent and chaotic circumstances within which the NGOs operated. The short funding cycles and volatile nature of emergencies often prohibit a systematic and rigorous evaluation of either the impact or the monitoring of multiple agencies in the same setting.

#### Wider level of impact analysis

There is an increasing interest in impact analysis at higher levels and a 'system-wide approach to performance' [7]. Several initiatives and mechanisms, from donors and humanitarian agencies, are attempting to move beyond the project level and consider sectoral, multi-sectoral, or system-wide impacts. For example, one of the objectives of the SMART initiative is to enable judgements about the overall impact of the humanitarian effort. Another example is the Inter-agency Health Evaluations in Humanitarian Crises Initiative that proposes to establish inter-agency health programme reviews in order to find new ways of looking at health programme performance and its impact on the health of affected populations [34].

A number of issues must be considered with wider levels of impact assessment. First, there is no reason to think that the constraints encountered when measuring the impact of particular interventions are erased when looking at a wider level. A particular difficulty is that of aggregation. The wider the level is, the more aggregation impact data require. Clearly, a donor or an aid agency looking at the overall effectiveness of its aid over a number of years needs far more aggregation than the evaluation of the impact of a single project conducted by a single agency. Finally, wider levels of impact assessment also generate new problems such as that of responsibility. Who is responsible for the collective impact of a number of individual humanitarian projects? Projects may have a positive impact taken individually, but the overall humanitarian effort may be insufficient compared with the level of needs. Who will account for the overall success or failure (if that is in fact possible to measure) of the humanitarian enterprise? This is a typical question that came out of the system-wide Rwanda evaluation. There is also a need for consensus in the relief community about the fundamental objective of health programmes.

## Conclusion

Despite existing efforts to improve the quality, accountability and performance of humanitarian aid in the health sector and more broadly, this paper has shown that there is limited knowledge about the health impact of humanitarian aid. The epidemiological tools potentially useful for analysing the impact of aid programmes are seldom used. As a result, humanitarian efforts rest on a limited evidence base. This is in large part due to lack of epidemiological skills found within NGOs working on the ground. Addressing this skills deficit will be essential if the rigour of routine assessments is to be improved.

In the current practice, the health impact of programmes is too often assumed rather than demonstrated. This is largely due to the use of performance or process indicators as proxy for impact, without the necessary evidence that the intervention is robustly linked with a health outcome. There needs to be a consensus regarding which types of intervention (measles immunisation, assuring that people have enough food and water) are linked to good health and the levels of service that are sufficient, in order to document that aid money is well spent. Further research on the links between particular interventions and health outcomes is required to build up this evidence base.

Efforts to document project impact should be woven into monitoring and surveillance activities, not only to reduce costs, but as a tool to improve program quality. The absence of systematic monitoring and surveillance in the humanitarian sector is a serious obstacle to assessing the impact of humanitarian aid. All too often an assessment of the impact is considered as a separate activity that takes place at the end of a project. The question of impact must be included throughout the project cycle, from the formulation of objectives to the final evaluation.

For health impacts to be more widely documented there needs to be adequately trained, experienced, and motivated staff present at the design and evaluation phases of projects. Part of the solution is increased funding for training and retaining staff who can act as a resource, but there must be also an increased collaboration between donors and relief workers that develops a culture rewarding the documentation of programme failures as well as successes as learning opportunities. While initiatives such as SMART provide a potentially useful platform for analysing the global impact of humanitarian aid, there is a risk that the efforts will focus exclusively on technical discussions regardless of the wider political dimension of humanitarian aid. Some agencies also fear that these mechanisms will reinforce the donor control over humanitarian agencies, instead of solely aiming to increase the quality and performance of humanitarian aid. Nonetheless, increasing accountability in all sectors of international aid and increasing expectations for the wellbeing of the world's downtrodden will eventually demand consistent and widespread documentation of humanitarian benefits. Existing epidemiological techniques can adequately do so, if only they were employed. The challenge will be to make this documentation occur through positive self-improvement motives rather than as a reactive response to criticism.

## Authors contributions

LR, a field epidemiologist, wrote the theoretical parts of the paper and conducted the review of reports of 15 health-related programmes; CAH, a researcher and humanitarian worker, wrote the sections that relate to humanitarian assistance and the practice of impact assessment. This paper is based on a research project into the impact of humanitarian aid carried out by the Humanitarian Policy Group at the Overseas Development Institute.

## Competing interests

The author(s) declare that they have no competing interests.
